# Synthesis of Copper Oxide-Based Nanoformulations of Etoricoxib and Montelukast and Their Evaluation through Analgesic, Anti-Inflammatory, Anti-Pyretic, and Acute Toxicity Activities

**DOI:** 10.3390/molecules27041433

**Published:** 2022-02-21

**Authors:** Sulaiman Sulaiman, Shabir Ahmad, Syeda Sohaila Naz, Sara Qaisar, Sayyar Muhammad, Amal Alotaibi, Riaz Ullah

**Affiliations:** 1 Department of Chemistry, Islamia College University, Peshawar 25120, Khyber Pakhtunkhwa, Pakistan; sulmankkpp@gmail.com (S.S.); shabir.ahmad@icp.edu.pk (S.A.); sayyar@icp.edu.pk (S.M.); 2 Nanosciences and Technology Department, National Centre for Physics, Quaid-i-Azam University Campus, Islamabad 44000, Punjab, Pakistan; syedasohailanaz@yahoo.com (S.S.N.); sara.qaisar@ncp.edu.pk (S.Q.); 3 Department of Basic Science, College of Medicine, Princess Nourah Bint Abdulrahman University, P.O. Box 84428, Riyadh 11671, Saudi Arabia; amaalotaibi@pnu.edu.sa; 4 Department of Pharmacognosy (MAPPRC), College of Pharmacy, King Saud University, Riyadh 11451, Saudi Arabia

**Keywords:** etoricoxib, montelukast, CuO nanoparticles, nanomedicine, analgesic potential, anti-pyretic agent, anti-inflammatory agent

## Abstract

Copper oxide nanoparticles (CuO NPs) were synthesized through the coprecipitation method and used as nanocarriers for etoricoxib (selective COX-2 inhibitor drug) and montelukast (leukotriene product inhibitor drug) in combination therapy. The CuO NPs, free drugs, and nanoformulations were investigated through UV/Vis spectroscopy, FTIR spectroscopy, XRD, SEM, and DLS. SEM imaging showed agglomerated nanorods of CuO NPs of about 87 nm size. The CE1, CE2, and CE6 nanoformulations were investigated through DLS, and their particle sizes were 271, 258, and 254 nm, respectively. The nanoformulations were evaluated through in vitro anti-inflammatory activity, in vivo anti-inflammatory activity, in vivo analgesic activity, in vivo anti-pyretic activity, and in vivo acute toxicity activity. In vivo activities were performed on albino mice. BSA denaturation was highly inhibited by CE1, CE2, and CE6 as compared to other nanoformulations in the in vitro anti-inflammatory activity. The in vivo bioactivities showed that low doses (5 mg/kg) of nanoformulations were more potent than high doses (10 and 20 mg/kg) of free drugs in the inhibition of pain, fever, and inflammation. Lastly, CE2 was more potent than that of other nanoformulations.

## 1. Introduction

Nanotechnology is a field of research in which substances with one dimension less than 100 nm are studied [1]. The origin of nanotechnology can be traced back to the famous lecture of Nobel laureate Richard P. Feynman (1959) in which he stated that ‘‘There’s Plenty of Room at the Bottom” [2,3]. Nanotechnology has brought revolutionary changes in the field of science, technology, agriculture, energy, health, and other industrializations. These changes are related to the vast behaviors of nanoparticles [4]. Copper oxide nanoparticles (CuO NPs) have a large surface area and unique morphological structures which impart them with characteristic physical and chemical properties. They are widely applicable in nanomedicines [5], PN junction diodes [6], humidity sensing [7], lithium-ion batteries [8], organic synthesis [9], antimicrobial activity, and biomedicine. CuO NPs show anti-bacterial and other anti-microbial activities [10].

Non-steroidal anti-inflammatory drugs (NSAIDs) are the most widely prescribed drugs for the treatment of pain, fever, and inflammation worldwide [11]. Acetylsalicylic acid was the first NSAID that was synthesized by Felix Hoffman in 1897 [12]. In 2004, more than 111 million NSAIDs were prescribed in the USA. Moreover, the number of prescriptions of NSAIDs in Europe is 7.7% [13]. These drugs are mostly prescribed to patients at or above the age of 65 years. These drugs are effective for pain, fever, and inflammation in osteoarthritis, psoriatic arthritis, injury, gout, and rheumatoid arthritis [14].

The primary cause of pain, fever, and inflammation is the production of the cyclooxygenase (COX) enzyme in the body of various organisms [15]. The basic function of NSAIDs is to stop the production of COX. The blockage of COX leads to the inhibition of the conversion of arachidonic acid to prostacyclin which is the cause of pain, fever, and inflammation in the body [16,17,18]. Although NSAIDs are effective against several diseases, they may also lead to some side effects such as gastrointestinal bleeding, renal failure, and heart diseases. The cause of inflammation and pain is from COX-2 enzymes and leukotriene products but most NSAIDs block both COX-1 and COX-2 enzymes [19]. However, COX-1 is important (via thromboxane) for the protection of the stomach [20]. The inhibition of COX-1 enzymes leads to gastric and heart problems [20,21]. Although some selective COX-2 inhibitors are available on the market, their high doses and bulk form lead to severe side effects [22,23]. The potency of drugs is enhanced with targeted drug delivery tools such as soft materials involved in biological and artificial membranes [24], PEG [25], alginate, and chitosan-coated nanoparticles [26]. These tools also reduce drug side effects.

In this research, efforts were made to synthesize nanoformulations of selective inhibition of COX-2 and leukotriene products. Therefore, etoricoxib (ET) was selected as a COX-2 inhibitor, which is mostly prescribed for the treatment of pain and arthritic conditions such as rheumatoid arthritis and osteoarthritis [27], and montelukast (MT) was used as a leukotriene product inhibitor drug. CuO NPs were used as nanocarriers for ET and MT in combination therapy. CuO NPs also possess potency as antioxidant and anticancer agents [28]. They show antibacterial activity against both Gram-positive and Gram-negative bacteria [29]. The nanosize of copper oxide provides a large surface area for the interaction of drugs and enhances its potency. Low doses of nanodrugs are more potent due to their large surface area and small size than bulk drugs. Nanoparticle-mediated drug delivery enhances the potency, solubility, and bioavailability of drugs [30,31], while free drugs are less soluble and have low bioavailability [32], and their high dose is administrated which increases the risk of side effects [33]. In this combination therapy, two drugs were loaded onto CuO NPs for the treatment of inflammation, fever, and pain. CuO NPs are disinfectant [34], antifouling [35], antiviral [36], and antimicrobial agents [37]; therefore, they were selected as nanocarriers for ET and MT to inhibit COX-2 and leukotriene products.

## 2. Results and Discussions

### 2.1. Structural Analysis

#### 2.1.1. UV/Vis Spectroscopy

**Figure 1:**
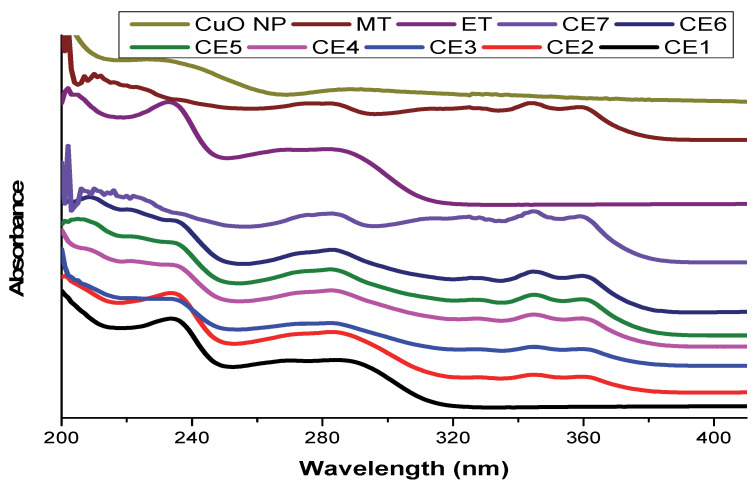
UV/vis spectrum of copper oxide nanoparticles (CuO NPs), etoricoxib (ET), montelukast (MT), and synthesized nanomaterials.

UV/Vis spectroscopy was used for the analysis of copper oxide nanoparticles, drugs, and synthesized nanomaterials (Figure 1). UV/Vis spectroscopy is a significant tool that is used to investigate the nature of bonds, bandgap calculations, and surface plasmon resonances. The UV/Vis spectrum was performed in the range of 200–800 nm. Solutions of CuO NP, ET, MT, and synthesized nanomaterials were prepared in ethanol. A characteristic peak of CuO NPs was observed at 290 nm, which confirmed its synthesis [38,39]. A characteristic peak of ET was observed at 233 nm which corresponds to the literature [40]; while that of MT was observed at 284, 324, 343, and 360 nm [41]. The characteristic peak of copper oxide nanoparticles disappeared in the nanoformulation, which indicates the adsorption of drugs. The peak of that drug was more prominent in nanoformulations where its ratio was higher. The peaks of MT were prominent in CE5, CE6, and CE7; while those of ET were prominent in CE1, CE2, and CE3. 

#### 2.1.2. FTIR Spectroscopy 

**Figure 2:**
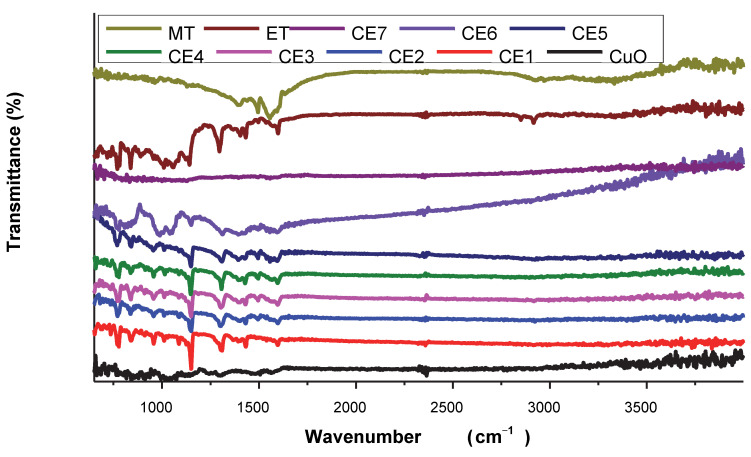
FT-IR spectrum of CuO NPs, ET, MT, and synthesized nanomaterials.

The synthesis of CuO NPs was also confirmed from FTIR analysis (Figure 2). The synthesis of CuO NPs was confirmed by the appearance of the characteristic peaks of CuO NPs in specific regions. The vibrational mode of CuO NPs was revealed in the range of 500–700 cm^−1^ by the three absorption peaks. The stretching of Cu-O was indicated by the peak at 525 cm^−1^. The synthesis of CuO NPs was confirmed from the peaks at 525 cm^−1^ and 602 cm^−1^ [42,43]. Characteristic peaks of ET were observed at 772, 830, 1010, 1140 cm^−1^ (corresponding to sulphone groups (−S=O)) [44], 1598.9 cm^−1^ (C=N stretching vibration), 839.0, 781.1, and 638.0 cm^−1^ (C–Cl stretching vibration) [45,46,47]. The characteristic peaks of MT were observed at 3396 (COOH stretching), 2980 (aromatic C–H stretching), 2925 (aliphatic C–H stretching), 1610 (CC stretching), 1595 (CN stretching), 1497 (aliphatic C–H bending), 1132 (C–O stretching), 1068 (aromatic C–Cl stretching), 837 (aromatic C–H bending), and 697 (C–S stretching) [48]. Although a few of the ET and MT peaks disappeared in nanoformulations, most of them appeared which shows their adsorption onto CuO NPs.

#### 2.1.3. X-ray Diffractometry

**Figure 3:**
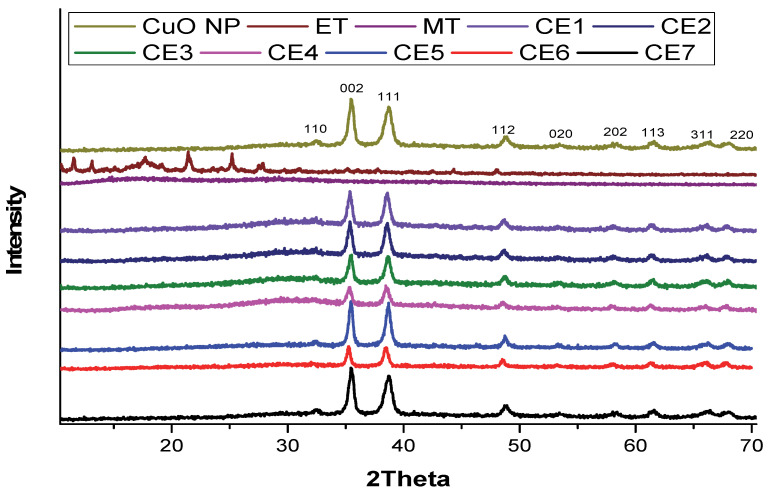
XRD spectrum of CuO NPs, ET, MT, and synthesized nanomaterials.

The synthesis of CuO NPs was confirmed from UV/vis spectroscopy and FTIR spectroscopy, but it was further matched with JCPDS cards for XRD analysis. X-ray diffractometry (XRD) (Figure 3) was conducted by using a D8 advance Bruker X-ray diffractometer (Bruker Germany) at NCP Islamabad, Pakistan. Characteristic peaks of CuO NPs were observed at 2θ = 32.44°, 35.47°, 38.62°, 48.86°, 53.45°, 58.06°, 61.60°, 66.45°, and 68.17° in the XRD spectrum which corresponded to 110, 002, 111, 112, 020, 202,113, 311, and 220 planes of the Miller’s indices, respectively [49]. The highly crystalline nature of the synthesized CuO NPs was confirmed from the sharp and well-defined reflections of the CuO NPs (JCPDS card no. 01-080-0076) [50]. CuO NPs have a monoclinic structure with a crystallite size of 13.7 nm. Characteristic peaks of ET were observed at 2θ = 11.9, 13.3, 16.5, 16.6, 18.2, 20.2, 22.8, 24.2, 26.5, 28.7, 29.4, 30.3, 32.5, 32.9, 34.8, 36.1, 39.1, 40.1, 41.2, 42, 42.9, 45.7, 47.6, 49.3, and 53.2 [51]. These peaks were masked or disappeared in the nanoformulations. The disappearance of peaks in the nanoformulations shows that ET was attached or converted to an amorphous form. MT has no characteristic peak in the XRD spectrum due to its amorphous nature. The crystallite structure of CuO NPs, ET, and nanoformulations was calculated through the Scherrer formula (Table 1).

#### 2.1.4. Scanning Electron Microscopy

**Figure 4:**
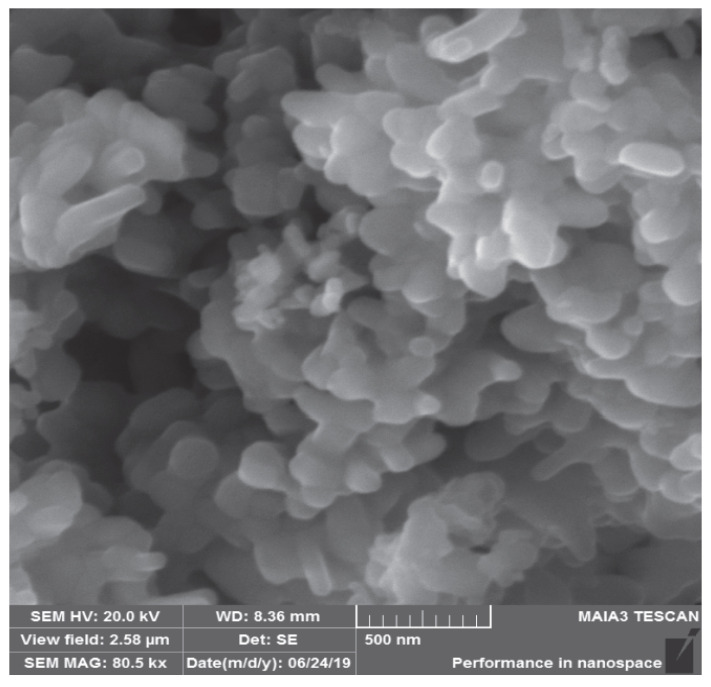
SEM image of CuO NPs.

The synthesized CuO NPs were investigated through SEM which showed agglomerated nanorods of CuO NPs of about 87 nm size (Figure 4).

#### 2.1.5. Particle Size Analysis

**Figure 5:**
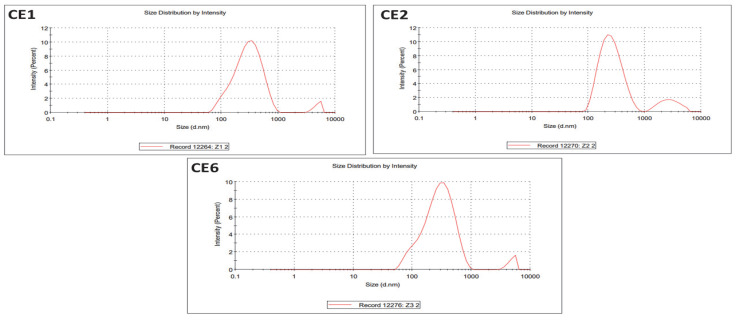
DLS graphs of CE1, CE2, and CE6.

The sizes of CE1, CE2, and CE6 were investigated through dynamic light scattering (DLS). The particle sizes of CE1, CE2, and CE6 were 271, 258, and 254 nm, respectively (Figure 5).

#### 2.1.6. Quantification of ET and MT

**Figure 6:**
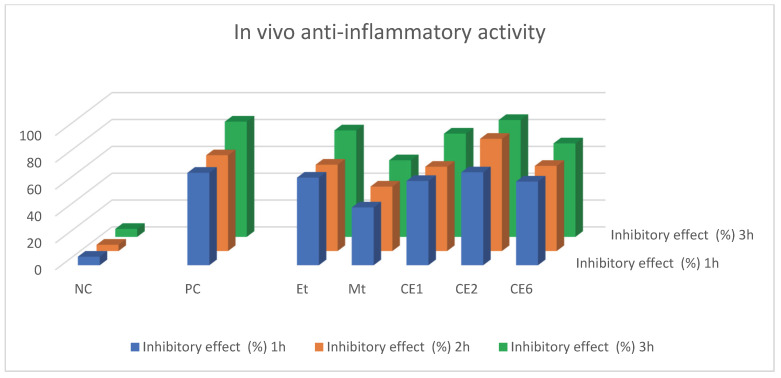
Graphical representation of in vivo anti-inflammatory activity. PC—positive control, NC—negative control.

The quantity or concentration of ET and MT in each nanoformulation was calculated by UV/Vis spectroscopy at a specific wavelength. The wavelength for quantification of ET was 235 nm while that of MT was 345 nm. However, the best wavelength for both drugs was 282 nm (Figure 6). The absorbance of samples followed Beer–Lambert’s law.

The quantities of ET and MT in 5 mg of each nanoformulations are given in Table 2. The drug loading efficiency was about 70–75%, calculated through UV analysis by using the following Equation (1) (encapsulation efficiency (EE)):

(1) $$\mathrm{EE}\;\%=\frac{\left(\mathrm{Total}\;\mathrm{drug}\;\mathrm{added}\right)-\left(\mathrm{non}-\mathrm{entrapped}\;\mathrm{drug}\right)\;}{\mathrm{Total}\;\mathrm{drug}\;\mathrm{added}}\times 100$$

##### Calibration Curve

The quantity of ET and MT in different nanoformulations with a mass of 5 mg was analyzed which shows a linear plot on the calibration curve. The results are given in Table 2.

##### Specificity

There was no interference from CuO nanoparticles in the nanoformulation of ET and MT.

##### Validation of Method

The performance of the system was precise and suitable during the entire analysis of the nanoformulations of ET and MT. Validation of the analysis method for the quantification protocol is reported in the literature both for HPLC and UV/Vis spectroscopy [52].

### 2.2. Bioactivities Studies

#### 2.2.1. In Vitro Anti-Inflammatory Activity

The method of inhibition of BSA denaturation was used for in vitro anti-inflammatory activity. It is obvious that the denaturing of proteins leads to inflammation in the body. In this method, solutions of samples were added to a solution of BSA and were then incubated at 37 and 55 °C. The % inhibition of BSA denaturation was investigated through UV/Vis spectroscopy at a fixed wavelength of 660 nm. The % inhibitory effect was calculated using the following Equation (2):

(2) $$\begin{array}{c} \%\;\mathrm{inhibition}\\ of\;denaturing \end{array}=\frac{\left(\mathrm{absorbance}\;\mathrm{of}\;\mathrm{control}\right)-\left(\mathrm{absorbance}\;\mathrm{of}\;\mathrm{sample}\right)}{\mathrm{absorbance}\;\mathrm{of}\;\mathrm{control}}\times 100$$

BSA denaturation was not inhibited in the negative control; therefore, inhibition of nanoformulations and standards was compared with it. Albeit the inhibitory effect of all nanoformulations was high; however, that of CE1, CE2, and CE6 was very high. Therefore, these nanoformulations were selected for further evaluation through in vivo studies. The results of % inhibition of BSA denaturation through nanoformulations are given in Table 3 [53].

#### 2.2.2. In Vivo Anti-Inflammatory Activity

In vivo anti-inflammatory activity was carried out through the carrageenan-induced hind paw edema method on albino mice at the Department of pharmacy, Quaid e Azam University, Islamabad. Mice of both sexes were used. Mice were treated with nanoformulations at a dose of 5 mg/kg body weight while doses of drugs were 10 mg/kg body weight through i.p. injection. They were treated with 0.1 mL of carrageenan 1% solution through the left hind paw and the paw volume was measured using a Plethysmometer. The % inhibition of inflammation was estimated from the decrease in the volume of the hind paw of mice. The % inhibition of inflammation was calculated using the following Equation (3) [54,55]:

(3) $$\%\;\mathrm{inhibition}=\frac{\left(V_{\mathrm{control}}-V_{\mathrm{test}}\right)}{V_{\mathrm{control}}}\times 100$$

The inhibition of inflammation was low in the first hour but increased in the second and third hour. The % inhibitory effect of CE2 was high against inflammation, which is shown in Table 4 and Figure 6.

#### 2.2.3. In Vivo Analgesic Activity

**Figure 7:**
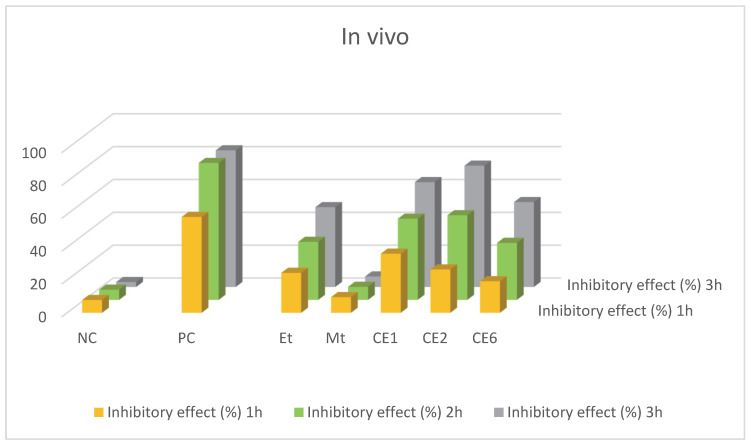
Graphical representation of in vivo analgesic activity.

In vivo analgesic activity was performed in albino mice through the hot plate method. Mice were treated with doses of nanoformulations intraperitonially. They were placed on a hot plate at 55 °C for a 30 s cut-off time. The body movements of mice were keenly observed. The % inhibition of pain was measured from the time they tolerate on the hot plate. If pain is more inhibited, the mice will spend more time on the hot plate and vice versa. The % pain inhibition of CE2 was very high as compared to other nanoformulations which is shown in Table 5 and Figure 7.

#### 2.2.4. In Vivo Anti Pyretic Activity

**Figure 8:**
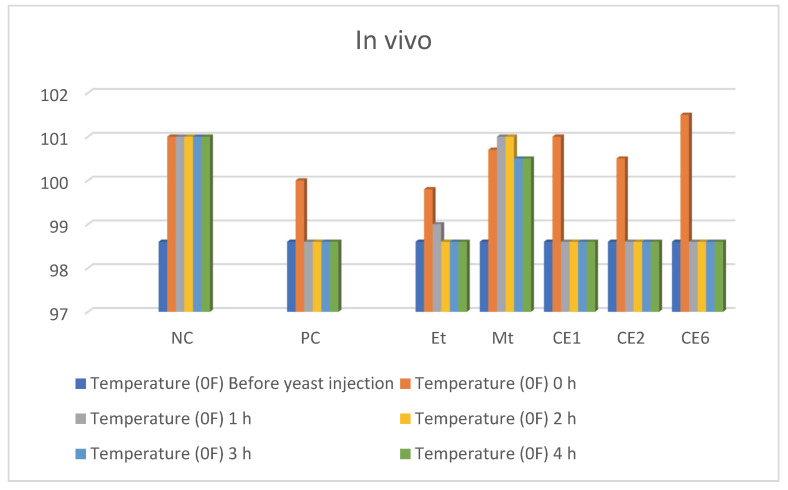
Graphical representation of in vivo anti-pyretic activity.

In vivo anti-pyretic activity was carried out in albino mice through the yeast-induced pyrexia method. To induce pyrexia, 10 mL/kg of 20% yeast solution was intraperitoneally injected into mice. The body temperature of mice was measured before the treatment of yeast solution through a lubricated thermometer. Mice whose body temperature increased by 0.6 °C were selected for this activity. These mice were treated with nanoformulations intraperitoneally to inhibit pyrexia. The temperature of mice was measured for 4 h after each hour, which was controlled in the first hour by CE1, CE2, and CE3 as shown in Table 6. The body temperature of mice is graphically represented in Figure 8 before and after treatment of the samples. The blue color indicates mice with normal body temperature (98.6 °F) while the orange color indicates mice with a high body temperature due to the treatment of yeast. The other four colors indicate the body temperature of mice following the treatment of samples.

**Table 6:** In vivo anti-pyretic activity.

Drug	Dose (mg/kg)	Temperature (°F)					
		Before Yeast Injection	0 h	1 h	2 h	3 h	4 h
Negative control (normal saline)	-	98.6	101	101	101	101	101
Positive control (paracetamol)	20 [56]	98.6	100	98.6	98.6	98.6	98.6
Et	20	98.6	99.8	99	98.6	98.6	98.6
Mt	20 [54]	98.6	100.7	101	101	100.5	100.5
CE1	5	98.6	101	98.6	98.6	98.6	98.6
CE2	5	98.6	100.5	98.6	98.6	98.6	98.6
CE6	5	98.6	101.5	98.6	98.6	98.6	98.6

#### 2.2.5. In Vivo Acute Toxicity Activity

In vivo acute toxicity activity was carried out in albino mice through the median lethal dose (LD50) method. Different doses of nanoformulations were given to mice and observed for 24 h. The mice were normal when treated with doses of 5, 10, 25, 50, and 100 mg/kg body weight; however, died when the dose was increased to 200 mg/kg body weight. It was confirmed that the nanoformulations are acutely toxic at or above 200 mg/kg body weight.

## 3. Materials and Methods

### 3.1. Materials

Copper nitrate trihydrate (Cu(NO_3_)_2_.3H_2_O), sodium hydroxide (NaOH), ethanol, ET, poly ethylene glycol (PEG, 6000), and polyvinyl acetate (PVA) were obtained from Merck (Darmstadt, Germany). UV/Vis spectra were recorded through Perkin Elmer (Waltham, MA, USA), UV/Vis spectrometer, Lambda 25. The FT-IR spectrum was performed on a JASCO FT/IR-6600 spectrometer. X-ray diffractometry (XRD) was conducted by using a D8 advance Bruker X-ray diffractometer equipped with a Cu anode (Bruker, Bremen, Germany).

### 3.2. Synthesis of Copper Oxide Nanoparticles

CuO NPs were prepared through the coprecipitation method. Cu(NO_3_)_2_.3H_2_O was used as a precursor salt. Cu(NO_3_)_2_.3H_2_O salt was reduced to NaOH to prepare CuO NPs. A 100 mM solution of Cu(NO_3_)_2_.3H_2_O was prepared in 100 mL deionized water. PEG-6000 was used as a surfactant in this synthetic work. A 100 mM solution of NaOH was very slowly added to the solution of Cu(NO_3_)_2_.3H_2_O with constant stirring, which led to an increase in the pH of the solution. The addition of NaOH was stopped when the pH become basic. The appearance of blackish particles represented the synthesis of CuO NPs. The pH of the solution was maintained at pH 7 by washing with deionized water. The solution was filtered through Whatman filter paper and the residue was dried, ground, and then calcinated at 500 °C for 4 h [57].

### 3.3. Synthesis of Etoricoxib and Montelukast Conjugated CuO Nanomaterials (CE)

CuO NPs were used as nanocarriers for the ET and MT. Separate solutions of CuO NPs, ET, and MT were prepared in ethanol. The CuO NPs solution was sonicated for 1h to completely dissolve it. Different ratios of ET and MT solutions were added to the CuO NPs solution (Table 7). The solution was kept for 2 h post stirring. Then, the solution was sonicated for 2 h and then centrifuged at 6000 rpm for 1 h. The synthesized nanoformulations were dried at room temperature. Drug loading efficiency was 70–75%. PVA was used as an encapsulation agent for ET- and MT-conjugated CuO nanomaterials. Moreover, 2.5% PVA was added to the synthesized nanoformulation.

#### 3.3.1. Quantification Protocol

The objective of the spectroscopic quantification of ET and MT was to determine their quantity in the nanoformulations. The quantification of ET and MT was carried out both simultaneously as well as separately for each drug. Although this may be carried out through HPLC or UV/Vis spectroscopy, here the analysis was performed through UV/Vis spectroscopy in the range of 200–800 nm. Therefore, different solutions of ET and MT were prepared in ethanol. Different ratios of ET and MT were added in different solutions. Separate and combined solutions were prepared for the drugs. The quantity of drugs was analyzed through UV/Vis spectroscopy for each solution [58]. The wavelength for quantification of ET was 235 nm while that of MT was 345 nm. However, the best wavelength for both drugs was 282 nm. 

#### 3.3.2. Samples Preparation

Five milligrams of ET was added to 20 mL of ethanol and sonicated for 30 min for complete dissolution. Five milligrams of MT was also added to 20 mL of ethanol in another beaker and completely dissolved. Then, different ratios of ET and MT (1:3, 1.5:2.5, 2:2, 2.5:1.5, and 3:1) were added to different beakers containing ethanol as the solvent and were completely dissolved through sonication. These solutions were diluted and analyzed through UV/Vis spectroscopy.

Solutions of CuO NPs were also prepared in ethanol. Then, solutions of ET and MT of the above ratios were added to a solution of CuO NPs in separate beakers. Next, the solution was sonicated and centrifuged at 6000 rpm. The supernatant was separated for each solution and was analyzed through UV/Vis spectroscopy. The difference in the quantity of ET as well as of MT, before and after addition to CuO nanoparticles, was calculated from analyzing the UV/Vis spectra [59].

### 3.4. Characterization

The FTIR analysis was carried out using the JASCO FT/IR-6600 spectrometer at a range of 400–4000 cm^−1^. The samples were taken in solid powder form. For each sample, the values of % transmittance were obtained in the form of an FTIR spectrum. FTIR measures the vibrations of atoms, molecules, and functional groups which correspond to the matching frequency of the infrared beam. Therefore, the FTIR spectrum consists of the functional group region and fingerprint region [60]. The XRD spectra were performed through a D8 advance Bruker X-ray diffractometer (Bruker, Bremen, Germany) for the investigation of crystallite size, miller indices, and phase determination. The spectra were calculated in the range of 10–90 2θ. Samples were taken in solid powder form for XRD analysis. The spectra were matched with JCPDS cards for verification. In this analysis, the principle of Bragg’s law (2dsinθ = nλ) was followed [61]. The crystal structure was calculated through Scherrer’s formula (Ls = 0.92λ/βs cosθB). Zetasizer or dynamic light scattering (DLS) is the technique used for the determination of the size of particles in the suspension. DLS was performed in the Pharmacy Department, QAU, Islamabad. DLS can measure particle sizes ranging from 1 nm to 10 μm. The DLS analysis is based on the Brownian motion of particles. The Stokes–Einstein equation was used to calculate the particle size (D = kT/6rhR) [62]. Scanning electron microscopy (SEM) was used to investigate the size, shape, texture, and morphology of nanoparticles. SEM analysis was carried out at PINSTIC Islamabad. The samples were taken in solid powder form and investigated by SEM. The basic principle of SEM for the investigation and analysis of substances includes scanning under a beam of electrons. In SEM analysis, the range of voltage is about 30 keV.

### 3.5. Bioactivities

#### 3.5.1. In Vitro Anti-Inflammatory Activity

The primary cause of inflammation is the denaturing of proteins in the body. Therefore, for measurement of the potency of drugs and nanoformulations in vitro, anti-inflammatory activity was performed through the inhibition of the bovine serum albumin (BSA) denaturing method. In this method, 5 mg/mL solution of each sample was prepared in DMSO and that of BSA (1% solution) was prepared in deionized water. Moreover, a 2 mg/mL solution of Diclofenac sodium was used as a standard. Then, 0.9 mL of BSA solution was added to a 0.1 mL solution of each sample and the standard. The solutions were incubated at 37 °C for 20 min and then at 55 °C for a further 20 min. These samples were evaluated through UV/Vis spectroscopy at 660 nm [63].

#### 3.5.2. In Vivo Anti-Inflammatory Activity

Albino mice were obtained from the National Institute of Health, Islamabad, and an ethics approval letter was obtained from the ethics committee of Islamia College, Peshawar, Pakistan, for the proper handling and care of albino mice.

In vivo anti-inflammatory activity was performed on albino mice through the carrageenan-induced hind paw oedema method. The hind paw volume was measured before and after the treatment of carrageenan using a Plethysmometer (UGO BASILE 7140). Doses of samples were prepared in normal saline water and were administered through intraperitoneal (i.p.) injection as 5 mg/kg body weight. To induce swelling, 0.1 mL of 1% carrageenan solution was injected into the left hind paw of each mouse. The paw volume was measured after one hour of drug treatment. The drug was not given to the negative control group [64,65].

#### 3.5.3. In Vivo Analgesic Activity

In vivo analgesic activity was carried out in albino mice using the hot-plate method of Eddy and Leimbach (1953) [66]. Mice of both sexes were used in this activity. The average weight of mice was 26–28 g. Doses of nanoformulations were given through i.p. injection as 5 mg/kg body weight. Then, mice were placed on a hot plate for a 35 s cut-off temperature. The temperature of the hot plate was adjusted to 55 °C [67].

#### 3.5.4. In Vivo Anti Pyretic Activity

The yeast-induced pyrexia method was used for evaluating in vivo anti-pyretic activity in albino mice. To induce pyrexia, 20% of aqueous brewer’s yeast solution was subcutaneously injected into mice at a dose of 10 mL/kg body weight [68]. The body temperature of each mouse was measured through a rectal thermometer before the treatment of the yeast solution. After 18 h, the body temperature of mice was again measured. Mice whose body temperature increased by 0.6 °C were selected. Doses of nanoformulations and standards were given to each mouse through i.p. injection. The body temperature of mice was measured for 4 h [56].

#### 3.5.5. In Vivo Acute Toxicity Activity

In vivo acute toxicity activity was carried out in albino mice through the median lethal dose (LD50) method [69]. Different doses of nanoformulations were given to mice and were observed for 24 h. The doses were 5, 10, 20, 50, 100, and 200 mg/kg body weight. The purpose of this activity was to measure the maximum amount of nanoformulations that were safe for organisms.

#### 3.5.6. Statistical Analysis

The studied bioactivities data are expressed as the standard error mean (SEM). The mean ± standard deviation (SD) was calculated for the data by the Equation (4):

(4) $$\%\pm \mathrm{SEM}=\frac{\%\pm \mathrm{SD}}{\sqrt{3}}$$

## 4. Conclusions

CuO NPs were used as nanocarriers of ET (selective COX-2 inhibitor drug) and MT (leukotriene product inhibitor drug) in different ratios. CuO NPs were synthesized through the coprecipitation method and investigated using different tools. The synthesized nanoformulations were evaluated through in vitro and in vivo bioactivities which confirmed that these nanoformulations were more potent than those of parent drugs. The in vitro anti-inflammatory activity indicated that CE1, CE2, and CE6 are of high potency; therefore, these three nanoformulations were further evaluated through in vivo bioactivities. The in vivo bioactivities showed that low doses (5 mg/kg) of nanoformulations were more potent than that of high doses (10 and 20 mg/kg) of free drugs.

## Figures and Tables

**Table 1:** Crystallite size of the nanoformulations.

S.No.	Name/Code	Crystallite Size (nm)
1	CuO	13.7
2	ET	41.64
3	MT	Amorphous
4	CE1	19.17
5	CE2	17.87
6	CE3	20.97
7	CE4	22.53
8	CE5	19.60
9	CE6	21.98
10	CE7	18.88

**Table 2:** Quantity of etoricoxib and montelukast in 5 mg of each nanoformulation.

S.NO.	5 mg	Etoricoxib (mg)	Montelukast (mg)	RSD (±)
1.	CE1	2.8	-	0.07
2.	CE2	2.1	0.7	0.05
3.	CE3	1.80	0.77	0.08
4.	CE4	1.45	1.35	0.06
5.	CE5	0.77	1.75	0.07
6.	CE6	0.75	2.10	0.08
7.	CE7	-	2.95	0.06

**Table 3:** In vitro anti-inflammatory activity.

S.No.	Code	Absorbance at 660 nm	Inhibition (%)± SEM
1	Negative control (DMSO)	0.6900	0
2	CE1	0.2039	70.45 ± 1.12
3	CE2	0.1463	78.79 ± 1.56
4	CE3	0.3822	44.61 ± 1.12
5	CE4	0.7444	7.884 ± 1.25
6	CE5	0.6335	8.188 ± 1.46
7	CE6	0.2040	70.43 ± 1.52
8	CE7	0.2802	59.39 ± 1.14
9	Etoricoxib (ET)	0.0221	96.80 ± 1.36
10	Montelukast (MT)	0.1476	78.61 ± 1.95
11	CuO	0.1677	75.69 ± 1.80
12	Positive control (diclofenac sodium)	0.0755	89.06 ± 1.75

**Table 4:** In vivo anti-inflammatory activity.

Drug	Dose (mg/kg)	Inhibitory Effect (%) ± SEM		
		1 h	2 h	3 h
Negative control (normal saline)	-	6.37 ± 1.08	4.75 ± 1.03	5.93 ± 1.02
Positive control (diclofenac sodium)	10	68.84 ± 1.23	71.33 ± 1.12	85.78 ± 1.24
Et	10	65.19 ± 1.21	64.30 ± 1.53	79.26 ± 1.42
Mt	10	43 ± 1.24	48 ± 1.35	57 ± 1.45
CE1	5	62.72 ± 1.21	62.77 ± 1.24	76.84 ± 1.22
CE2	5	69.17 ± 1.25	83.56 ± 1.31	86.99 ± 1.32
CE6	5	62.29 ± 1.39	63.38 ± 1.42	69.59 ± 1.38

**Table 5:** In vivo analgesic activity.

Drug	Dose (mg/kg)	Inhibitory Effect (%) ± SEM		
		1 h	2 h	3 h
Negative control (normal saline)	-	7.79 ± 1.02	6.20 ± 1.10	3.03 ± 1.21
Positive control (diclofenac sodium)	10	58.34 ± 1.21	83.38 ± 1.24	83.22 ± 1.45
Et	10	24.30 ± 1.24	35.37 ± 1.10	48.61 ± 1.15
Mt	10 [54]	9.53 ± 1.32	7.95 ± 1.24	6.41 ± 1.42
CE1	5	35.97 ± 1.54	49.41 ± 1.41	63.86 ± 1.24
CE2	5	26.37 ± 1.45	51.53 ± 1.32	73.88 ± 1.52
CE6	5	19.19 ± 1.12	34.71 ± 1.21	51.72 ± 1.54

**Table 7:** Composition of nanoformulations.

S.No.	Code	CuO (%)	Etoricoxib (%)	Montelukast (%)	PVA wt%
1	CE1	20	80	-	2.5
2	CE2	20	60	20	
3	CE3	20	50	30	
4	CE4	20	40	40	
5	CE5	20	30	50	
6	CE6	20	20	60	
7	CE7	20	-	80	
8	ET	-	100	-	-
9	MT	-	-	100	-
10	CuO	100	-	-	-

## Data Availability

All available data incorporated in the MS.

## Sample Availability

Samples of the compounds are available with First author.
